# Physical Properties and In Vitro Biocompatible Evaluation of Silicone-Modified Polyurethane Nanofibers and Films

**DOI:** 10.3390/nano9030367

**Published:** 2019-03-05

**Authors:** Chuan Yin, Sélène Rozet, Rino Okamoto, Mikihisa Kondo, Yasushi Tamada, Toshihisa Tanaka, Hatsuhiko Hattori, Masaki Tanaka, Hiromasa Sato, Shota Iino

**Affiliations:** 1 Interdisciplinary Graduate School of Science and Technology, Shinshu University, 3-15-1, Tokida, Ueda-shi, Nagano 386-8567, Japan; yinkawa@outlook.com (C.Y.); selene.rozet@gmail.com (S.R.); j3992a1953m@gmail.com (R.O.); Kondo.mikihisa@exc.epson.co.jp (M.K.); ytamada@shinshu-u.ac.jp (Y.T.); 2 Silicone-Electronics Materials Research Center, Shin-Etsu Chemical Co., 1-10, Hitomi, Matsuida-Machi, Annaka-Shi, Gunma 379-0224, Japan; hhattori@shinetsu.jp (H.H.); m.tanaka@shinetsu.jp (M.T.); 3 Dainichiseika Color & Chemicals Mfg. Co., 1-4-3, Ukima, Kita-ku, Tokyo 115-8622, Japan; hrsato@daicolor.co.jp (H.S.); iino@daicolor.co.jp (S.I.)

**Keywords:** silicone modified polyurethane nanofibers, physical properties, cell attachment, cell proliferation, cytotoxicity

## Abstract

In this study, the physical properties and the biocompatibility of electrospun silicone-modified polyurethane (PUSX) nanofibers were discussed and compared with PUSX films. To investigate the effects of different structures on the physical properties, tensile strength, elongation at break, Young’s modulus, water retention, water contact angle (WCA) and thermal conductivity measurements were performed. To prove the in vitro biocompatibility of the materials, cell adhesion, cell proliferation, and cytotoxicity were studied by NIH3T3 mouse embryonic fibroblasts cells following by lactate dehydrogenase (LDH) analysis. As a conclusion, the mechanical properties, water retention, and WCA were proven to be able to be controlled and improved by adjusting the structure of PUSX. A higher hydrophobicity and lower thermal conductivity were found in PUSX nanofibers compared with polyurethane (PU) nanofibers and films. An in vitro biocompatibility evaluation shows that the cell proliferation can be performed on both PUSX nanofibers and films. However, within a short period, cells prefer to attach and entangle on PUSX nanofibers rather than PUSX films. PUSX nanofibers were proven to be a nontoxic alternative for PU nano-membranes or films in the biomedical field, because of the controllable physical properties and the biocompatibility.

## 1. Introduction

Electrospun nanofibers have been used in various fields such as filtration, catalysis, clothing, and biomedical applications, because of their submicron size and high surface area, along with their porous architecture [1,2]. Especially for biomedical applications, electrospun mats provide the lightness in weight, porosity, flexibility in technique, as well as a good support for cells to attach and grow. Their capacity to exchange nutrients and gases makes them suitable for tissue engineering, wound dressing, drug delivery, health care, etc. [3]. A non-woven matrix composed of nanofibers is easily produced via electrospinning, and it is architecturally similar to the nanofibrous structure of extracellular matrix [4]. If necessary, the nanofibers can be further functionalized via the incorporation of bioactive species (e.g. enzymes, DNAs, and growth factors) to better control the proliferation and differentiation of cells seeded on the scaffolds [5]. These attributes make electrospun nanofibers well-suited as scaffolds for tissue engineering.

Among various kinds of nanofibers, polyurethane nanofibers were selected as one of the most suitable choices for biomedical applications, thanks to the unique properties of polyurethane. Electrospun polyurethane nanofibers have been successfully used in wound dressing, due to an excellent oxygen permeability and barrier properties [6]. Water permeability is also important, as it keeps the wound moist and prevents the accumulation of fluid around the wound and on its cover. These covers perform a preventive function against infection with microorganisms, to absorb blood and wound fluids to contribute to the healing process, and in some cases, to apply medical treatment to the wound [7,8,9].

However, there are still several limitations and disadvantages of polyurethane nanofibers to be applied in the biomedical field, such as poor thermal capability, poor weatherability, and flammability. In order to improve the properties of polyurethane nanofibers, silicone groups were introduced into polyurethane polymer chains to fabricate silicone-modified polyurethane (PUSX) and to optimize the electrospinning parameters. In this study, PUSX nanofibers were evaluated by physical properties and cell culture studies, and they were compared with the films. The advantages of polyurethane, silicone, and nanofibers are very attractive for this work. This new material is expected to be applied in many fields as an improved alternative for polyurethane nanofibers, such as wound dressing and tissue engineering, due to the biocompatibility of silicone. Before going for the in vitro cell attachment and proliferation applications, all of the prepared nanofibers were analyzed in detail by various methods, and compared with films. To investigate the effects of different structure (block and graft type), chain lengths, and silicone concentration, evaluation of the physical properties was performed. Tensile tests were performed to investigate mechanical properties such as tensile strength, elongation at break, and Young’s modulus. The water contact angle (WCA) measurement and water retention tests were carried out to determine the hydrophobicity of the PUSX material. The thermal conductivity was analyzed in order to discuss the heat retention ability of the PUSX nanofibers and films. In order to reveal the potential for cell adhesion and proliferation, NIH3T3 mouse embryonic fibroblasts cells were cultured on all the samples, followed by lactate dehydrogenase (LDH) activity. The toxicity of the PUSX nanofibers and films were evaluated by using direct contact based on ISO 10993-5. Therefore, as the purpose of our research, the influence of PUSX structures on the physical properties and biocompatibility is investigated. PUSX nanofibers might be expected as an ideal alternative for PU nanofibrous membranes or films in the biomedical fields.

## 2. Experimental Section

### 2.1. Materials

**Figure 1:**
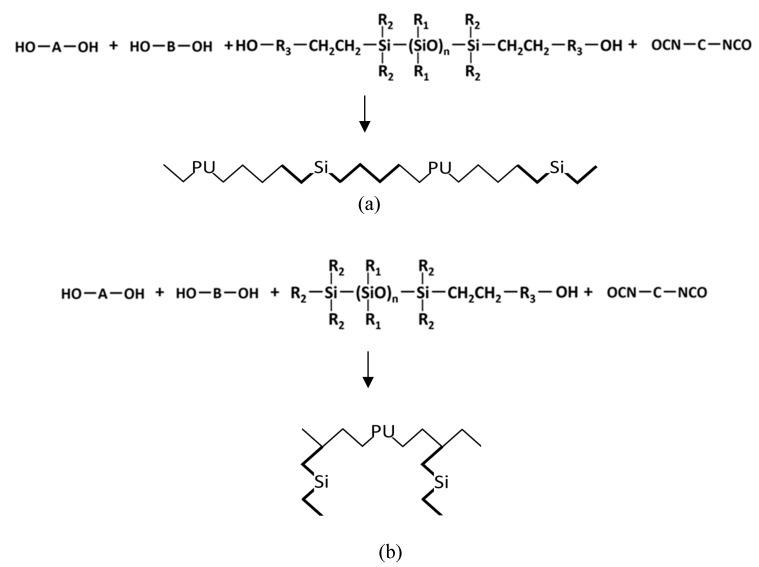
Structures of block-type silicone-modified polyurethane (PUSX) (**a**) and graft-type PUSX (**b**).

The 12 kinds of PUSX solutions and films were kindly synthesized and provided by Shin-Etsu Chemical Co., Ltd., and Dainichiseika Color & Chemicals Mfg. Co., Ltd. These synthesized PUSX solutions in *N*,*N*-dimethylformamide/ ethyl methyl ketone (DMF/MEK) standardized a solid content of 30 wt%. The structures of two types of PUSX are shown in Figure 1. PUSX with different silicone concentrations and chain lengths are listed in Table 1. Solvents such as *N*,*N*-dimethylformamide (DMF) and ethyl methyl ketone (MEK) were purchased from Wako Pure Chemical Industries., Ltd., and used as received.

### 2.2. Electrospinning Method of PUSX Nanofibers

All the electrospinning solutions were prepared by diluting the PUSX solutions (30 wt%) in DMF/MEK mixed solvent (v/v = 64:36), and stirring at room temperature for 48 h in order to obtain homogeneous solutions. All electrospinning experiments were performed at room temperature (22 °C) under the optimized parameters of our previous study [10], and the deposited nanofibers were collected on a moving metallic collector. A 10–20 kV voltage was applied while the needle tip-to-collector distance was 10 cm with an irradiation angle of 30°, and the air flow rate in the spinning environment was 0.1 mL/min. 

The surface morphology of the nanofibers was investigated by scanning electron microscope (SEM, JSM-6010LA JEOL, Tokyo, Japan) at an accelerating voltage of 10 kV. Before SEM analysis, the prepared samples were coated by using a platinum sputter coater (Ion sputter JFC-1600 JEOL, Ltd, Tokyo, Japan) under 30 mA for 60 s. The diameters of the nanofibers were measured by ImageJ (National Institutes of Health, Bethesda, MD, USA). The average fiber diameters were calculated from data of at least 50 measurements per sample.

### 2.3. Physical Properties

Tensile tests were performed by a compact tabletop universal tensile tester (EZTest/EZ-S, Shimadzu Corporation, Kyoto, Japan) for samples 10 mm long and 5 mm wide, at a crosshead speed of 10 mm/min. At least 10 specimens were tested for each sample. To compare the mechanical properties of the PUSX nanofibers with PUSX films, the same tests were performed on the PUSX films.

Thermal conductivities were determined by a KES-F7 Thermal LaboⅡB precision rapid thermal property measurement unit (KES-F7 Thermal Labo, Kato Tech Co., Ltd, Kyoto, Japan). The temperature of the water box was set to room temperature. Samples (5 × 5 cm^2^) were then placed on the water box, and the heat plate of the bottom temperature box (B. T. box) was placed on the upper surface of the samples. After reaching a constant value, the heat flow loss W (watts) of the B.T. was recorded using a panel meter. Steady heat flow lost was calculated as the following equation:

W = K·A·△T/D

where D is the thickness of the samples (cm), A is the area of the B.T. heat plate (cm^2^), △T is the temperature difference of sample (°C), and K is the thermal conductivity. The thermal conductivity K was calculated by the following equation:

K = W·D/A△T (W/cm °C) = 100 W·D/A△T(W/mK).

While taking measurements with the B. T. Box, the applied pressure could be adjusted. The standard value was set as 6 g/cm^2^. The temperature of the B. T. Box heat plate was controlled with an error of less than 0.1 °C.

Water retention tests were performed based on JIS L 1913 6.9.2. The electrospun PUSX nanofibers, and films of dimensions 100 × 100 mm^2^ were incubated in distilled water for a period of 15 min, and then weighed. Water retention capacity was determined as the increase in the weight of the fibers. The percentage of water absorption was calculated as in the following equation:

m = (m2 − m1)/m1 × 100%

where m2 and m1 are the weights of the samples in wet and dry environments, respectively.

WCA is an easy measurement for determining the wettability of the materials by a liquid. The static contact angle of pure water for the surfaces of the PUSX samples was measured by an automated contact angle meter (DM-501Hi, Kyowa Interface Science Co., Ltd, Saitama, Japan) after randomly dripping 2 μL of purified water on the surfaces of the samples. The droplets on the samples were captured after 1000 ms through an image analyzer, and the WCA, θ, was calculated by the software through analyzing the shape of the drop. When depositing a droplet onto the material, the water will form a droplet shape. The point where the surface, the water, and the vapor meet, is called the three-face point, and it determines the contact angle. The relationship between the contact angle, the surface free energy, the liquid surface tension, and the interfacial tension between solid and liquid is defined by the Young equation:

γS = γL cosθ + γSL,

where θ is the contact angle, γL is the surface free energy of the solid, and γSL is the interfacial tension between the solid and liquid.

Usually, when the WCA is less than 90°, the material can be considered to be hydrophilic while the material is hydrophobic. It is worth mentioning that if the WCA is between 150° and 180°, it shows the high water-repellency of the material.

### 2.4. In Vitro Biocompatible Evaluation

#### 2.4.1. Cell Culture Studies

Before using the samples for in vitro cell culture, it is essential to remove the DMF/MEK mixed solvents because of the possible cytotoxicity. All of the samples were washed with distilled water for 48 h and dried in oven at 80 °C overnight. Then, the nanofibers and films were cut into round shapes with a diameter of 10 mm, with three replicates per sample prepared. Sterilization was performed by deeply soaking the samples in 70% ethanol aqueous solution in a multi-well tissue culture polystyrene (TCPS) dish for 1 h, followed by rinsing three times in phosphate buffer saline (PBS) to remove all traces of ethanol.

NIH3T3 mouse embryonic fibroblasts were used to measure the cell adhesion and proliferation. For the cell adhesion test, 50,000 cells (in 1 mL of medium) were mixed well into the sample. After 3 h, the cells were harvested in 1 mL of 0.5% Triton X-100/PBS solution, and evaluated by LDH assay for adhesion evaluation of the cells.

The cell proliferation test was a quantitative investigation of the capacity for the cells to grow on the electrospun nanofibers and films. At the seeding step of the proliferation culture, 5000 cells were added into the sample. The experiment lasted for a total of seven days, and the results of the first, third, fifth, and seventh days were compared.

The LDH activity was immediately measured by ultraviolet absorption at a wavelength of 340 nm, using the Thermo Scientific Multiskan FC microplate photometer (Thermo Fisher Scientific Inc., Waltham, MA, USA) with a recorder. The enzyme activity of LDH can be measure from chemical reaction of LDH, when it is released into the cells’ medium from the damaged or dead cells, due to cell membrane damage. LDH converts lactate using NAD as a coenzyme, and produces pyruvic acid and NADH. The number of cells was calculated from the calibration curve obtained by the relation between the known number of cells, and the absorbance value at 340 nm of NADH in the assay supernatant.

The shape of the cells was observed by SEM to qualitatively investigate the cell reactions when in contact with the electrospun nanofibers. After each incubation period, the sample was fixed with paraformaldehyde (PFA) as a cross-linking fixation agent, to stop the proliferation of cells, and to preserve their shapes. The sample was further dehydrated by serially using ethanol gradient solutions of 50, 70, 95, and 99.5% for 30 min each, over a continuous process. Then, the sample was coated with platinum for SEM analysis.

#### 2.4.2. Toxicity Evaluation

The toxicities of the PUSX nanofibers and films were evaluated by using direct contact based on ISO 10993-5. Briefly, cells were seeded evenly over the surface of each plate and incubated at 37 °C until the cells covered the whole surface. The samples were then placed on the cell layers in the center of the plates, and the culture medium was replaced. In order to determine the toxicity in accordance with Grade 0 (nontoxic) to Grade 4 (severe toxic) evaluation, Trypan blue was added after 24 h in each plate, and observed by morphological changes. This is the most obvious and direct way to reflect the impact of testing the materials on the cell [11].

### 2.5. Statistical Analysis

Significance in vitro biocompatibility and physical properties were statistically analyzed by a one-way analysis of variance (ANOVA) using R free software. Statistical significance was set at *p* < 0.05 to identify which groups were significantly different from the other groups.

## 3. Results and Discussions

### 3.1. Morphology of PUSX Nanofibers Prepared for Physical and Biocompatability Evaluations

In the previous study [10], we successfully prepared 12 kinds of PUSX nanofibers under the optimized conditions, and investigated the effects of solvents and solution concentrations on the as-spun nanofibers, along with the morphological appearance.

Representative SEM images of PUSX nanofibers are shown in Table 2. It can be seen that the surface morphology of the block-type PUSX was smooth and continuous, with fiber diameters ranging from 400 nm to 720 nm. The mean diameters decreased with the increase of both the chain lengths of silicone, and the silicone concentration. The surface morphology of graft-type PUSX nanofibers was also fine and continuous, with the mean diameters ranging from 460 nm to 560 nm. Compared with block-type PUSX, graft-type PUSX nanofibers did not show a clear influence of the chain length on the electrospinning parameters. Compared with the PU nanofibers, the PUSX nanofibers showed a more uniform surface with a smaller diameter, due to the hydrophobicity of the silicone group. In this study, the physical properties and biocompatibilities of the prepared samples are discussed.

### 3.2. Mechanical Properties

#### 3.2.1. Tensile Strength

**Figure 2:**
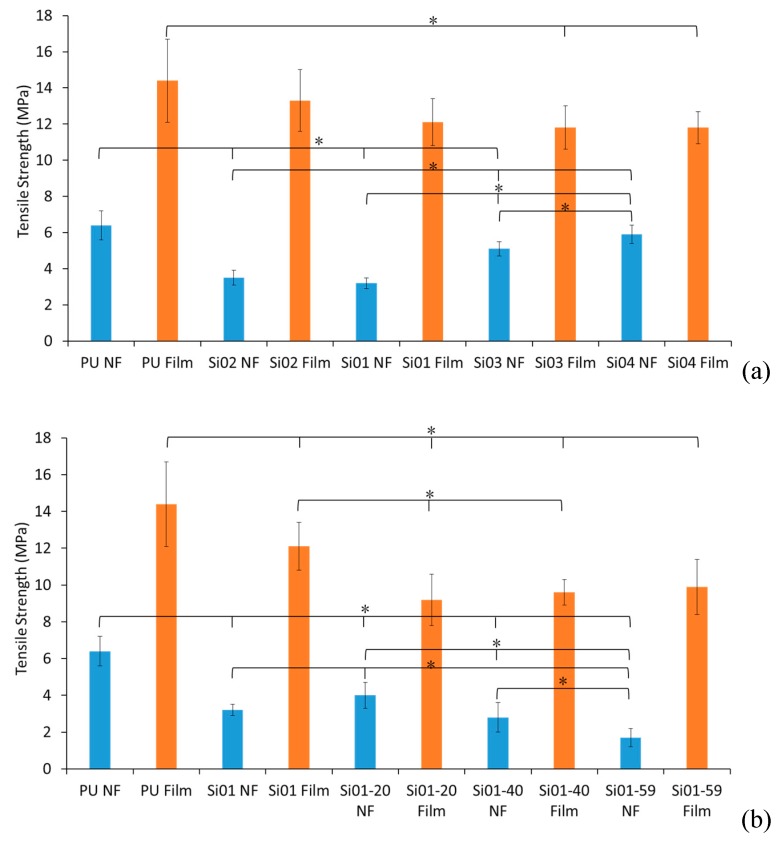

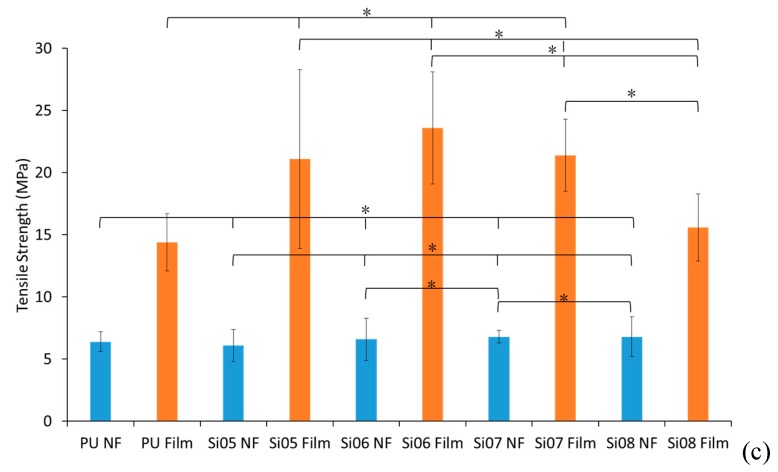
Comparison of tensile strength (MPa). “*” was statistically significant (*p* < 0.05) between each pair of samples. (**a**) Block-type PUSX nanofibers and films with various chain lengths, (**b**) Block-type PUSX nanofibers and films with various silicone concentrations, (**c**) Graft-type PUSX nanofibers and films with various chain lengths.

Figure 2a–c shows the graphs of the trend of tensile strength, and a comparison of all the samples. Referring to all three graphs, the PUSX films had better tensile strength than the nanofibers, because of the porosity and the fiber orientation of the nanofibers. Due to the porosity of the nanofibers, the cross-sectional area was apparent, compared to the cross-sectional area of the films, which made the tensile strength supposedly higher than the results obtained. Pure PU nanofibers had the highest strength because of the high mechanical properties of PU. Figure 2a,b show that for block-type PUSX nanofibers, the tensile strength increased with an increase of silicone chain length, and Si04 samples with the longest silicone chain length (n = 50) showed the highest tensile strength of 5.9 MPa. Samples with longer silicone chain lengths showed smaller diameters and a more improved orientation of the molecular chains while being prepared under the optimized parameters, which contributed to the higher tensile strength. Meanwhile, the tensile strength decreased with the increase of silicone concentration, because of the low cohesive force of the silicone structure, and the decreasing concentration of PU.

When the silicone concentration increased, the low cohesive force changed the tensile strength of the material. On the other hand, the increase of silicone concentration caused the decrease of the ratio of PU in the polymer, and the weight percent of PU as well, which means there was a lack of the higher mechanical structure (PU). The tensile strengths of the films did not show much statistical significance because of the random orientation of the molecular chains.

In the graph of graft-type PUSX nanofibers and films (Figure 2c), Si08 nanofibers showed the highest tensile strength, at 6.8 MPa, with the Si05 sample showing the lowest result of 6.1 MPa. There was almost no difference in the observed trends, because the results were in the same range.

In this case, the silicone groups on the side chain were not able to influence the properties much, because the tensile strength was mainly determined by the high mechanical properties of PU in the main chain.

**Figure 3:**
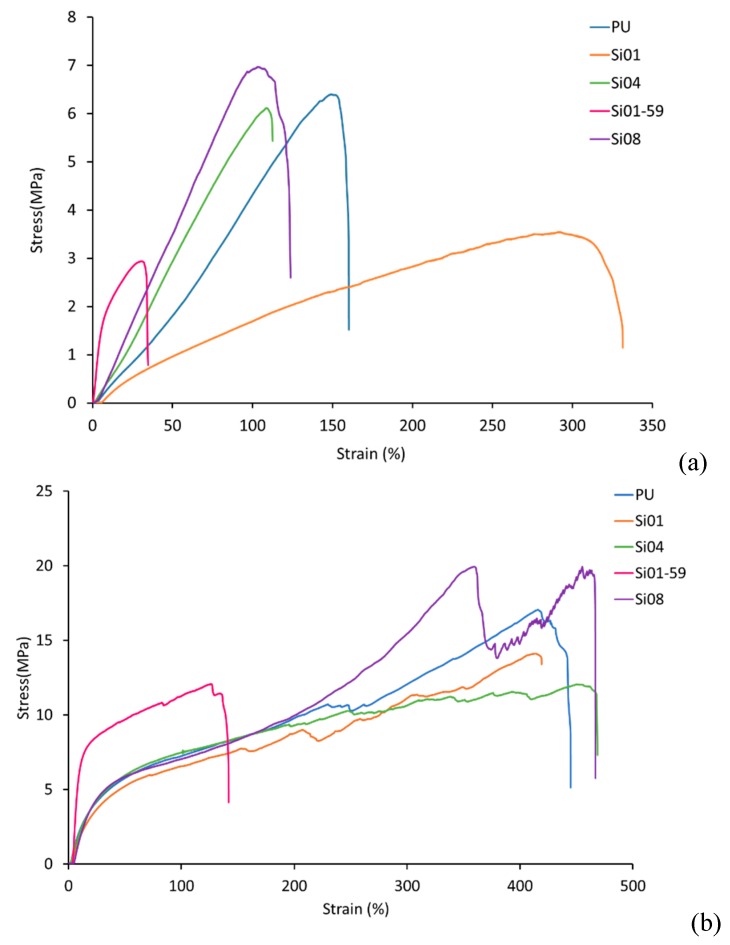
Comparison of stress–strain curves. (**a**) Stress–strain curves of the PUSX nanofibers, (**b**) Stress–strain curves of the PUSX films.

The tensile stress–strain curves of the electrospun PUSX nanofibers and the PUSX films are shown in Figure 3. Typical curves, each from a different structure of PUSX, were plotted for an obvious comparison. From Figure 3a, the differences before and after silicone modification could be easily observed. Si01-59 samples, with the highest silicone concentration in the block structure, show the lowest mechanical performances out of the fiber membranes, because of the low cohesive force of silicone. Instead, the tensile stress–strain curves of PUSX films showed very similar trends and much better mechanical performances, because of the random orientation of the molecular chains.

#### 3.2.2. Elongation at Break

**Figure 4:**
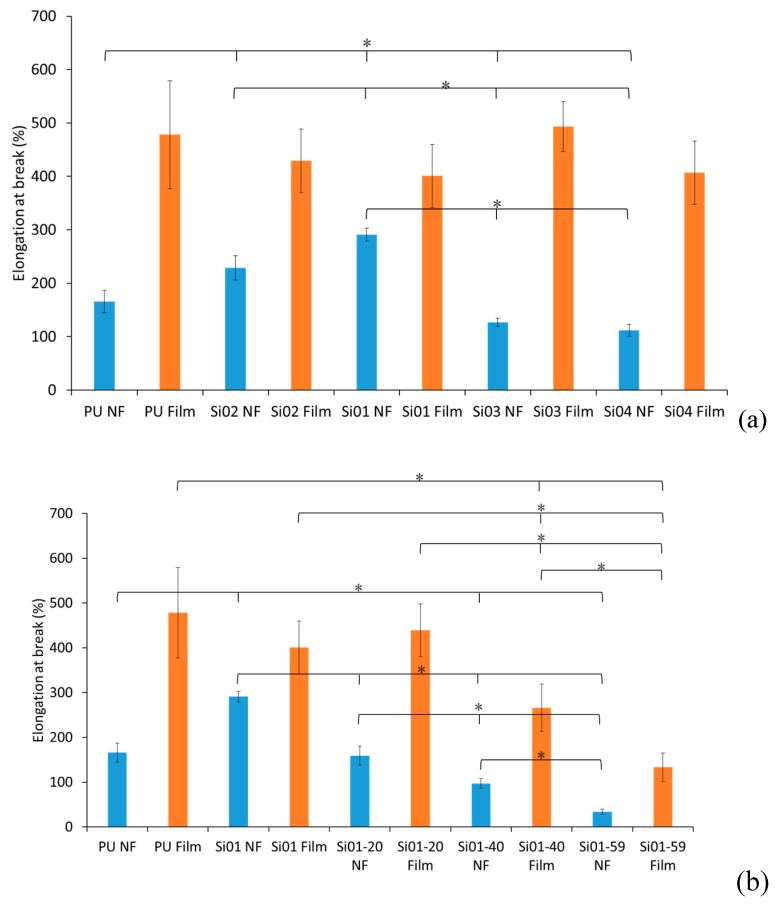

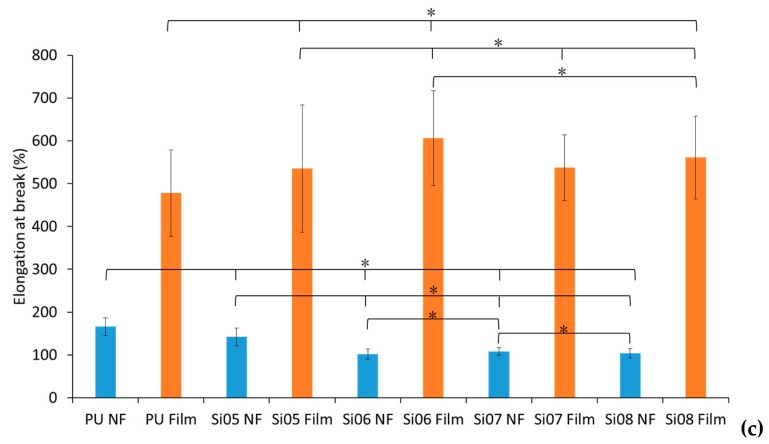
Comparison of elongation at break (%). “*” was statistically significant (*p* < 0.05) between each pair of samples. (**a**) Block-type PUSX nanofibers and films with various chain lengths, (**b**) Block-type PUSX nanofibers and films with various silicone concentrations, (**c**) Graft-type PUSX nanofibers and films with various chain lengths.

Referring to Figure 4, the elongation at break decreased with the increase of both silicone chain length and silicone concentration in block-type PUSX nanofibers. For the PUSX nanofibers with different silicone chain length, the increase of silicone chain length caused a decrease in fiber diameters under optimized conditions, which lead to the increase of entanglement and frictional resistance in the nanofibers. For PUSX nanofibers with different silicone concentrations, the increase of silicone concentration resulted in the decrease of polyurethane concentration in the molecular chains, and the high elongation property of polyurethane became difficult to observe. Meanwhile, the elongation at break of the graft-type PUSX nanofibers showed very similar results to each other for the same reason as with the tensile strength. As for the graft type, the elongation at break did not show an obvious trend, as the silicone groups on the side chains are not able to influence the nanofiber properties because the ratio of polyurethane and silicone in the molecular chain do not change with an increase of chain length. PUSX films have better tensile strengths than nanofibers, because of the porosity and fiber orientation of the nanofibers.

#### 3.2.3. Young’s Modulus

**Figure 5:**
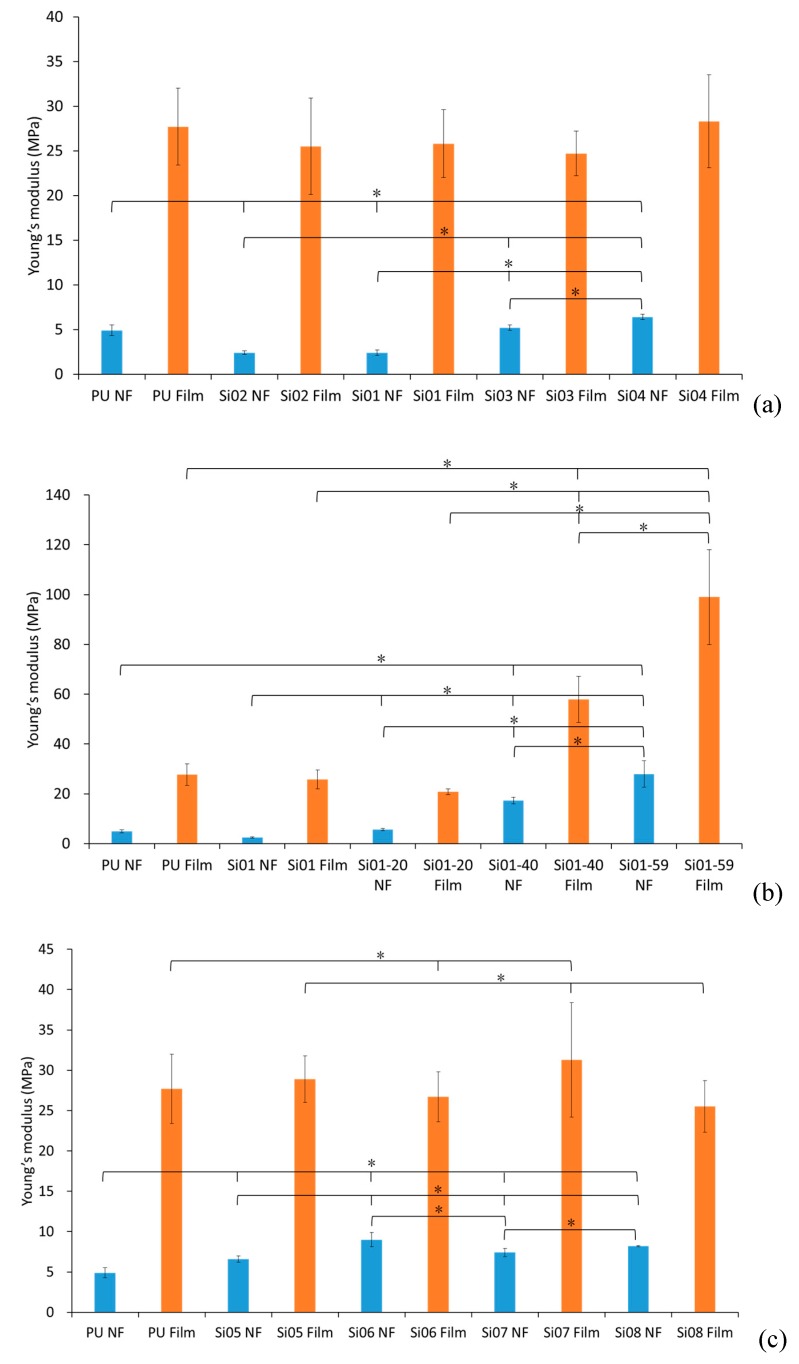
Comparison of Young’s modulus (MPa). “*” was statistically significant (*p* < 0.05) between each pair of samples. (**a**) Block-type PUSX nanofibers and films with various chain lengths, (**b**) Block-type PUSX nanofibers and films with various silicone concentrations, (**c**) Graft-type PUSX nanofibers and films with various chain lengths.

Referring to Figure 5, the Young’s modulus increased with the increase of both the chain length and the silicone concentration in block-type PUSX nanofibers. As the silicone chain lengths and concentrations increased, the concentration of PU became lower and lower, and the characteristics of PU (with a high elongation at break) became difficult to observe, which meant that the samples were more elastic. Moreover, the existence of silicone also made it more difficult to change the shapes of the samples.

For both block- and graft-type PUSX nanofibers, all of the tensile test results showed that PUSX films have higher tensile strengths than nanofibers. This phenomenon can be explained by the orientation of the polymer chain, and the different structures of the nanofibers and films (porosity and density).

### 3.3. Thermal Conductivity

**Figure 6:**
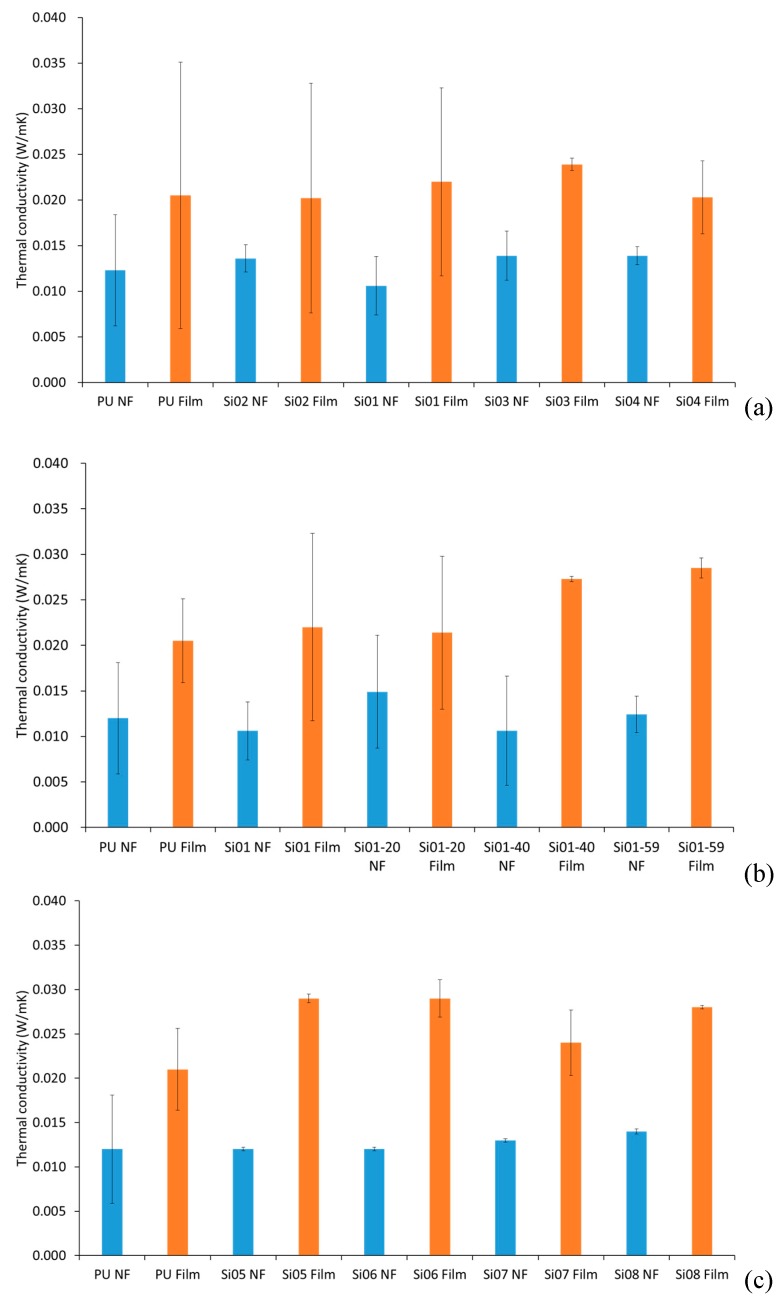
Comparison of the thermal conductivity (W/mK). (**a**) Block-type PUSX nanofibers and films with various chain lengths, (**b**) Block-type PUSX nanofibers and films with various silicone concentrations, (**c**) Graft-type PUSX nanofibers and films with various chain lengths.

Figure 6 shows the comparison of the thermal conductivity between block-type PUSX nanofibers and films and graft-type PUSX nanofibers and films. For the thermal conductivity analysis, there was no obvious trend of varying chemical structures such as varying silicone chain lengths, varying silicone concentrations and block or graft structures of PUSX. The thermal conductivity was influenced mostly by the shapes of the samples. Both block- and graft-type PUSX nanofibers have much lower conductivity than films, because of the pores keeping the air inside. The heat insulating property is proved here. This result can be explained by the heat-retaining property of nanofibers because of the high porosity.

It is worth mentioning that in solid objects, heat is transferred via conduction. For electrospun structures, much of the volume is made of empty spaces between fibers. Usually, the conduction along the fiber as part of the whole volume is negligible. But if the fibers becomes too compact, the packing density will be increased beyond an optimum standard, heat transfer via conduction through the solid fiber becomes significant, and the overall insulation property will be decreased. The insulation properties of the electrospun nanofibers are made more controllable due to their packing density compared to the films, to meet the demands of the market. As an ideal alternative to PUSX films, PUSX nanofibers provides good heat insulating properties and lightness in weight.

### 3.4. Water Retention and Water Contact Angle

#### 3.4.1. Water Retention

In Table 3, there is a huge gap between the pure PU and PUSX nanofibers where the PU nanofibers showed the highest water retention of 280%, with statistical significance (*p* < 0.05).

Block-type PUSX nanofibers showed a decreasing trend in water retention with the increase of both silicone chain length and silicone concentration, because of the hydrophobicity of silicone. This also means that the water repellency of the PU nanofibers are able to be improved by introducing the silicone groups into the main chain. Meanwhile, compared with films, since nanofibers have a high porosity for holding water, the water retentions of the nanofibers are higher than films. Both the PU nanofibers and films have the highest water retentions. Higher hydrophobicity of silicone structure and moisture permeability were proven.

Meanwhile, for graft-type PUSX nanofibers, the silicone groups on the side-chain are not able to increase the hydrophobicity of the material, so that the water retention did not show any difference between the PU nanofibers and the graft-type PUSX nanofibers.

#### 3.4.2. Water Contact Angle

**Figure 7:**
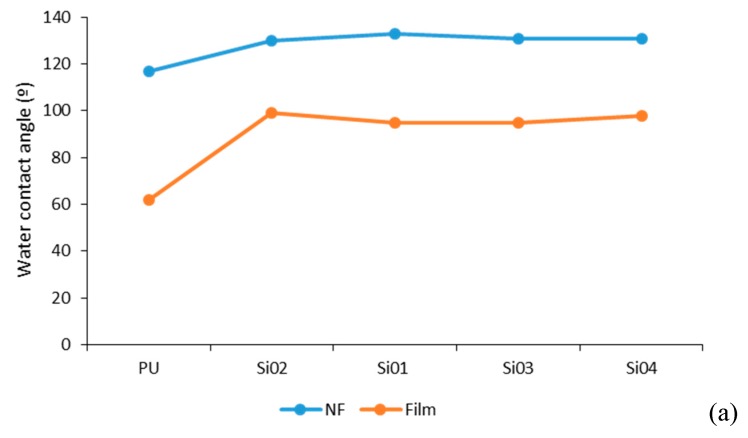

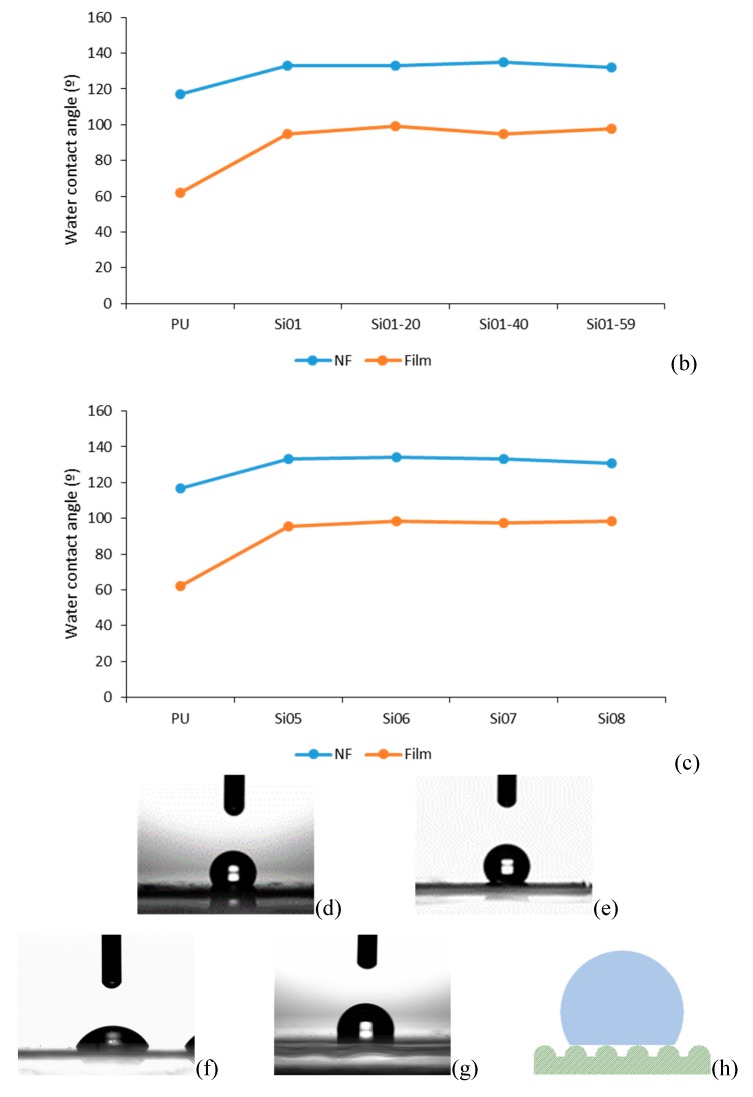
Comparison of water contact angle (WCA, degrees). (**a**) Block-type PUSX nanofibers and films with various chain lengths, (**b**) Block-type PUSX nanofibers and films with various silicone concentrations (**c**). WCA images of (**d**) PU nanofibers, (**e**) PUSX Si08 nanofibers, (**f**) PU films, (**g**) PUSX Si08 films, and (**h**) the Lotus microstructure (Cassie’s state).

Figure 7 shows that the water contact angles of PU were much lower than PUSX for both the nanofibers and films. This phenomenon might be caused by the high hydrophobicity of the silicone structure. It also proved the results of water retention when comparing PU and PUSX materials. The PUSX nanofibers showed higher WCA than the films, because the surface of the nonwoven nanofiber membrane was much rougher than the films. The pores of the nanofibers make the surface microstructure similar to the lotus structure of Cassie’s state (Figure 7h).

It is worth mentioning that for graft-type PUSX nanofibers, the water retention appeared to be higher compared with the block-type PUSX nanofibers, but the results of the water contact angle were similar. This might be caused by the different surfaces of the block-type and graft-type nanofibers. It is supposed that the silicone groups were distributed differently on the graft-type PUSX nanofibers than on the block-type PUSX nanofibers.

### 3.5. Cell Culture Studies

#### 3.5.1. Cell Attachment and Cell Proliferation

**Figure 8:**
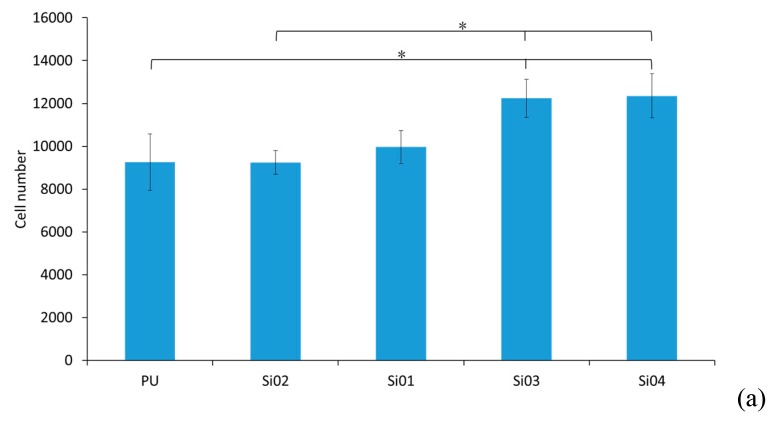

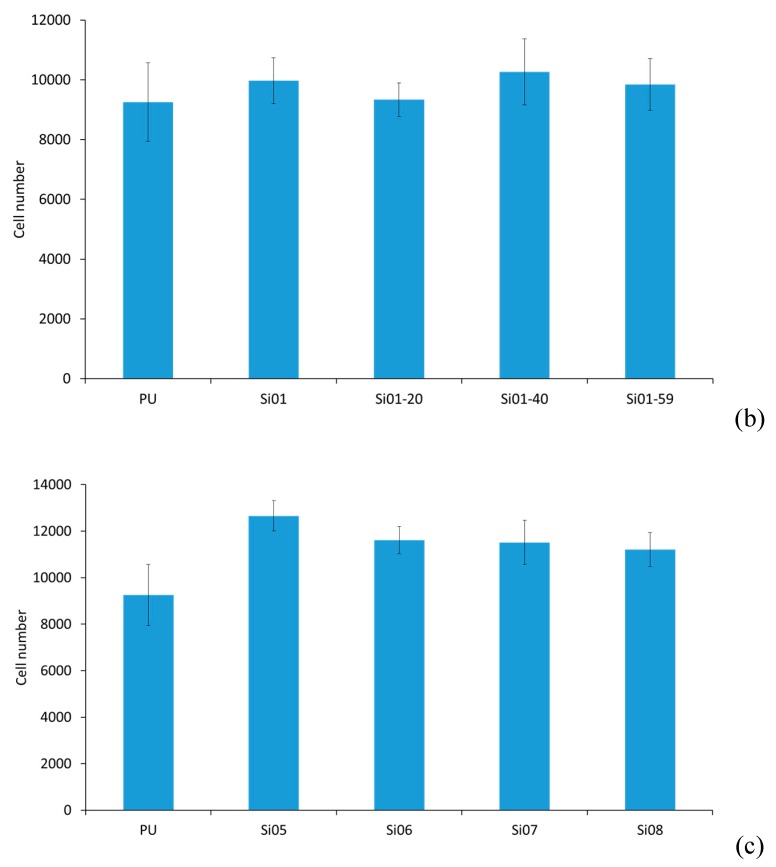
The attachment of NIH3T3 cells, (**a**) Block-type PUSX nanofibers with various chain lengths, (**b**) Block-type PUSX nanofibers with various silicone concentrations, (**c**) Graft-type PUSX nanofibers after cells have attached for three hours. “*” was statistically significant (*p* < 0.05) between each pair of samples.

To investigate the in vitro biocompatibility of the blended nanofibers, NIH3T3 cells were cultivated on to PUSX nanofibers and films of different structures. Cell attachment results were obtained and calculated after 3 h, and are showed in Figure 8. As the result, the number of adhered cells on block-type PUSX nanofibers became higher with the increase of silicone chain length. The reason for the increase of the fibroblast cells might be due to NIH3T3 cells being easily grown on the surface, which has a higher water repellency and hydrophobic surface characteristics. As discussed in Section 3.4, a higher water repellency is found with the increase of silicone chain length in block-type PUSX nanofibers. The cell attachment results of the PUSX films were not shown, due to the very low numbers after 3 h. Cells take a shorter time to adhere on nanofibers than on films, because of the porosity of the electrospun nanofibers. For cell attachment, PUSX nanofibers turned out to be more suitable than films.

**Figure 9:**
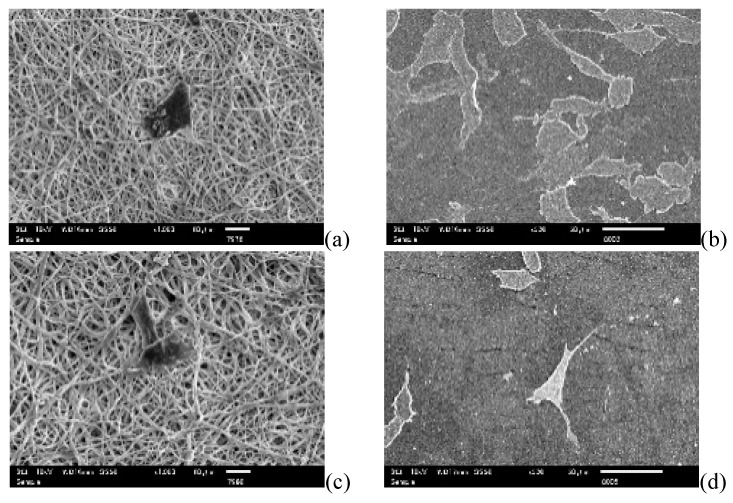

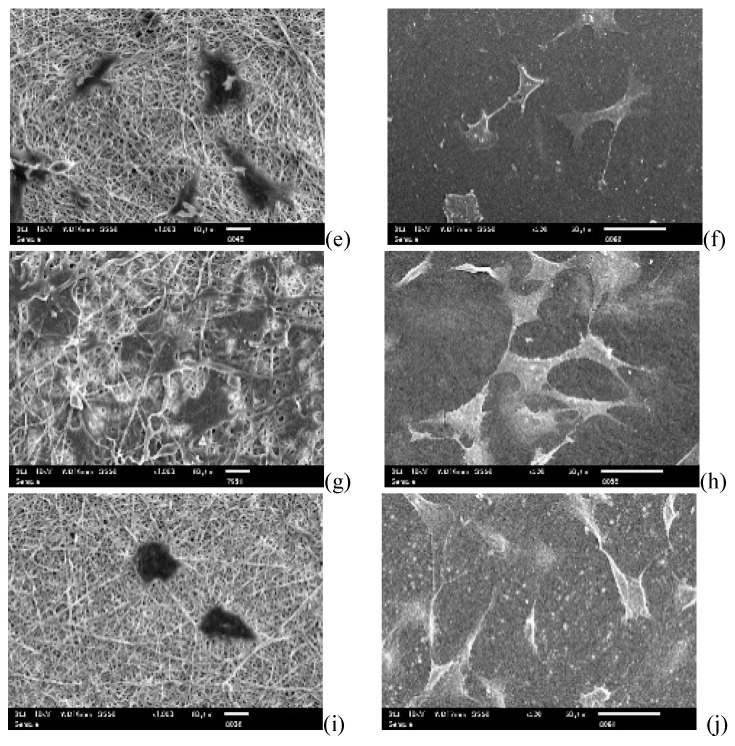
SEM images of NIH3T3 cells after culturing for three days on each sample. Cell attachment morphologies on PU nanofibers (**a**) and films (**b**), Si01 nanofibers (**c**) and films (**d**), Si01-59 nanofibers (**e**) and films (**f**), Si04 nanofibers (**g**) and films (**h**), Si08 nanofibers (**i**) and films (**j**). (Magnification of nanofibers 1000×, films: 500×).

Figure 9 represents the SEM images of the NIH3T3 fibroblast cells cultured for three days on different PUSX nanofibers and films with different structures. It can be seen that after three days of culture, there were more cells on the films than on the nanofibers, but the entanglement of the cells was totally different. The cells attached in the pores of the nanofibrous membranes with rough surfaces were much easier to manage as a scaffold for tissue engineering. Their stability was much higher than the cells attached onto the surfaces of the films. The reason might be that nanofibers have a fiber diameter of 400–700 nm, mimicking the extracellular matrix (ECM), as well as pores that help the cells to stay stable in the membranes. This work suggested that the PUSX nanofibers have an important advantage of being able to physically biomimic the natural ECM for tissue engineering applications, and cell ingrowth and cell encapsulation in the nanofibrous scaffolds are equally important. The architecture of a scaffold and the material used to play an important role in modulating tissue growth and response behavior of the cells that have been cultured onto the scaffold. In this regard, the scaffold should not only work as a substrate for cell attachment, growth, and proliferation, but also facilitate cell migration, ingrowth, and assembly into a stereo-structure. Referring to the SEM morphologies, the cells could attach onto PUSX nanofibers better than onto films, because the porosity makes the nanofibers more stereo than in films.

**Figure 10:**
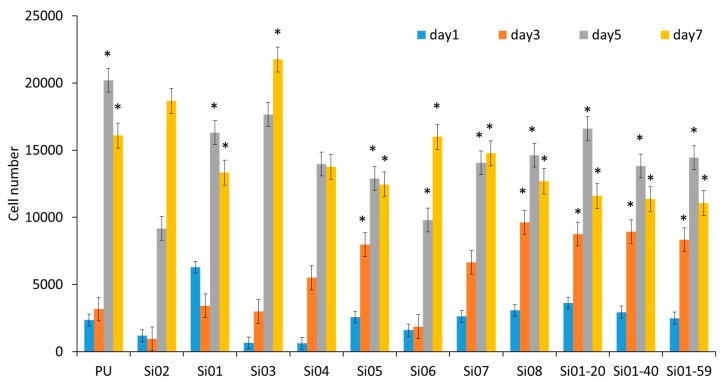
The proliferation of NIH3T3 cells on block-type PUSX nanofibers with various chain lengths, block-type PUSX nanofibers with various silicone concentrations, and graft-type PUSX nanofibers after cells were cultured for 1, 3, 5, and 7 days, respectively. “*” was statistically significant (*p* < 0.05) for each sample between 1 day and 3, 5, and 7 days.

Figure 10 shows the cell proliferation results after one day, three days, five days, and seven days, respectively. The doubling time of NIH3T3 in the normal cell culture condition was between 20 to 26 h, which means that it requires 30 h to produce 10,000 cells. However, the conditions of the nanofibers can slow the processes down, because the structure allows less space for the cell to adhere as quickly as on the normal substrate. From the results, all 12 kinds of PUSX nanofibers were proven to be appropriate for cell proliferation, with a maximum cell number of around more than 10,000 on the fifth day. PUSX nanofibers can be applied in the biomedical field as a better alternative to PU nanofibers, and with controllable physical properties, as seen in the similar results of the cell proliferation test. As a result, this study, it was confirmed that biomedical materials of desired physical properties are able to be prepared by changing the structure without losing the same level of biocompatibility.

#### 3.5.2. Toxicity Evaluation

**Figure 11:**
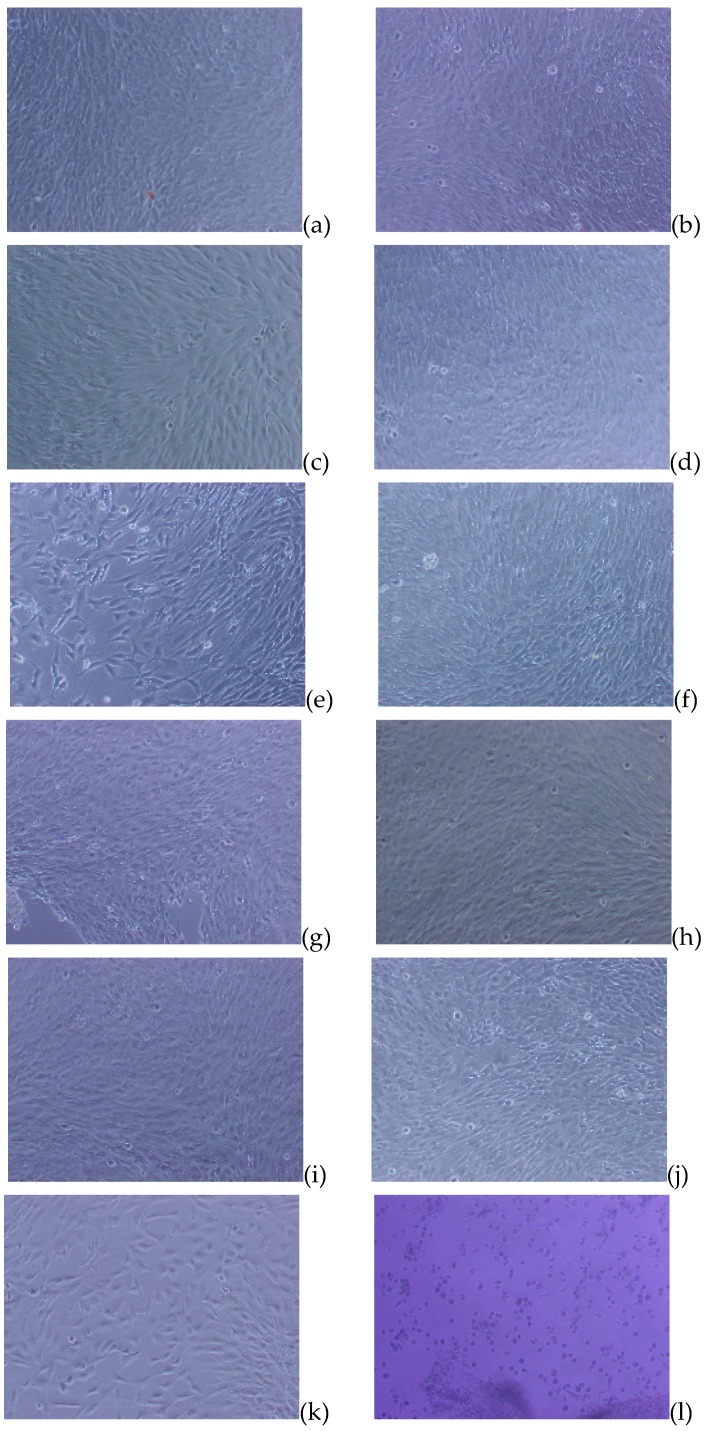
The toxicity evaluation of PU and PUSX samples. PU nanofibers (**a**) and films (**b**), Si01 nanofibers (**c**) and films (**d**), Si01-59 nanofibers (**e**) and films (**f**), Si04 nanofibers (**g**) and films (**h**), Si08 nanofibers (**i**) and films (**j**), compared to the negative control (**k**) and the positive control (**l**). (Magnification: 40×).

The results as displayed in Figure 11 showed that both the PUSX films and nanofibers caused less toxicity when they were in contact with cells. The cells were able to keep a spreading shape, and discrete intra-cytoplasmic granules with no cell lysis, which can be considered as a survival mechanism compared with the positive control (cells became round and layers were completely destroyed). The morphological grade of the cytotoxicity is supposed to be 0.

There was no obvious difference between the PUSX and PU materials, because of the biocompatibility of silicone. To the best of our knowledge, silicone has been extensively used in medical areas, in several products such as breast implants, contact lenses, lubricants, sealers, artificial cardiac tubes and valves, urethral and venous catheters, membranes for blood oxygenation, dialysis tubes, orthopedic applications, and facial reconstructions, because of its high biocompatibility [12,13]. The existence of the silicone group does not change the biocompatibility of the material. From these results, the PUSX materials showed a suitable biocompatibility for use as biomedical materials, such as waterproof bandages or scaffolds for tissue engineering.

Therefore, our novel nanofibrous membranes favored fibroblast cell attachment and growth by providing a stereo-structure environment that mimics the ECM, and which is considered to be biofriendly after toxicity evaluation. Indeed, there are a large amount of studies for developing biomedical materials for wound dressing and tissue engineering. For instance, Fenghua Xu et al. suggested tannic acid/chitosan/pullulan composite nanofibers, which showed synergistic antibacterial activity and the potential for deep and intricate wound healing. Compared with these materials, PUSX nanofibers show the controllable tensile strength of PU, which suggests potential applications for long-term tissue engineering, and hydrophobicity that is influenced by the silicone groups. As we know, wound infection is one of the main areas of concern in the management of the wound environment. Infection complicates treatment, and it impedes the healing process by damaging tissue, reducing wound tensile strength and inducing an undesirable inflammatory response [14,15,16]. PUSX nanofiber wound dressing may provide a hydrophobic effect, which helps to control bacteria by adsorbing bacteria onto the dressing surface. These results provide insight for the potential use of PUSX nanofibers for wound healing and tissue engineering into clinical practice in the future.

In the present study, the physical properties and biocompatible properties of PUSX nanofibers were investigated and compared with films. As a conclusion, for all of the analyses, physical properties, mechanical properties, water retention, and water contact angle (WCA) can be controlled and improved by adjusting the structure. Unfortunately, the graft-type PUSX did not show obvious changes in mechanical strength, because the side chains of silicone could not have as much influence as the main chain of PU does. Conversely, the graft-type PUSX nanofibers were the most similar alternatives to PU nanofibers in terms of mechanical properties, but with better water repellency according to the water contact angle results. Higher hydrophobicity and lower thermal conductivity were also found in the PUSX nanofibers, due to the unique advantages of nanofibers compared with films. This material can be expected to be applied in various fields. For instance, by controlling the silicone chain length and the concentration of block-type PUSX nanofibers, it can be applied in the medical field as bandages or scaffolds, the apparel field as outdoor goods and sportswear, and it can also be used for air or water filters.

In vitro biocompatible evaluation shows that cell proliferation can be performed on both the PUSX nanofibers and films. However, for cell attachment, the cells are not able to attach onto the PUSX films in a short time, nor entangle in the material, PUSX nanofibers were proven to be more appropriate for cell culture study. As we know, PU nanofibers have been developed as biomedical materials for several years. For instance, Lakshmi R. Lakshman et al. [17] and Afeesh R.Unnithan et al. [18] reported wound-dressing materials with antibacterial activity, which provided a basic understanding of the design for efficient PU nanofiber-based antibacterial wound dressing materials. Chang Hun Lee et al. [19] and Rui Chen et al. [20] demonstrated that electrospun PU nanofibers had the characteristics of a native extracellular matrix, and they may be used effectively as an alternative material for tissue engineering and functional biomaterials.

## 4. Conclusions

In this research, the physical properties and the biocompatibility of PUSX nanofibers were discussed and compared with PUSX films. Tensile strength increased with the increase of silicone chain length, and decreased with the increase of silicone concentration. The elongation at break decreased with the increase of both the silicone chain length and the silicone concentration in block-type PUSX nanofibers, while the Young’s modulus showed the entirely opposite trend. Higher hydrophobicity and lower thermal conductivity were found in PUSX nanofibers, compared with PU nanofibers and films. An in vitro biocompatible evaluation shows that PUSX nanofibers are able to be applied in the cell culture field, and that it shows very little toxicity. Compared with films, PUSX nanofibers shows better cell attachment over a short period of time. Therefore, electrospun PUSX nanofibers with highly controllable physical properties can be expected as ideal alternatives to PU membranes applications of the biomedical field, such as for wound dressings and tissue engineering.

## Figures and Tables

**Table 1:** Polyurethane (PU) samples and silicone modified polyurethane (PUSX) samples of block type and graft type.

Sample	PU	Si01	Si02	Si03	Si04	Si01-20	Si01-40	Si01-59	Si05	Si06	Si07	Si08
Molecular Structure	×	Block Type							Graft Type			
*M_w_* (×10^5^)	1.48	1.69	1.39	1.59	1.66	1.74	2.01	2.33	1.56	1.61	1.57	1.62
*M_n_* (×10^5^)	0.75	0.87	0.73	0.79	0.75	0.88	1.02	1.11	0.71	0.70	0.72	0.78
Silicone Concentration (wt%)	0	10	10	10	10	20	40	59	10	10	10	10
Silicone Chain Length (*n*)	×	20	10	30	50	20	20	20	10	25	30	120

**Table 2:** Scanning electron microscope (SEM) morphologies and average diameters of the PUSX nanofibers under the optimized electrospinning parameters (magnification: 2000×) [10].

Sample	PU	Si01	Si02	Si03
SEM	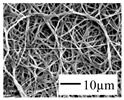	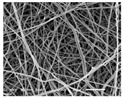	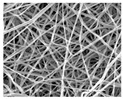	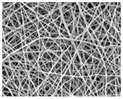
Average Diameter (nm)	720	636	690	548
Sample	Si04	Si01-20	Si01-40	Si01-59
SEM	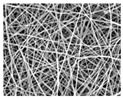	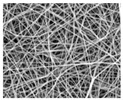	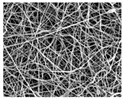	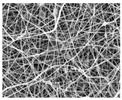
Average Diameter (nm)	440	531	402	471
Sample	Si05	Si06	Si07	Si08
SEM	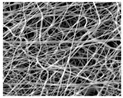	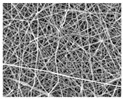	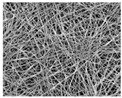	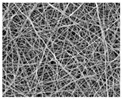
Average Diameter (nm)	564	544	456	456

**Table 3:** Comparisons of water retention.

Block-Type PUSX with Different Silicone Chain Lengths	PU	Si02	Si01	Si03	Si04
Nanofiber Water Retention (%)	280.0 ± 40.0	27.0 ± 11.0	19.0 ± 8.1	11.0 ± 4.1	12.0 ± 2.5
Film Water Retention (%)	2.3 ± 1.5	2.7 ± 1.1	1.5 ± 0.2	5.8 ± 5.0	3.7 ± 1.1
Block-type PUSX with Different Silicone Concentrations	PU	Si01	Si01-20	Si01-40	Si01-59
Nanofiber Water Retention (%)	280.0 ± 40.0	19.0 ± 8.1	4.7 ± 3.1	2.7 ± 0.9	6.9 ± 6.0
Film Water Retention (%)	2.3 ± 1.5	1.5 ± 0.2	3.7 ± 0.7	2.7 ± 1.0	2.9 ± 2.3
Graft-Type PUSX	PU	Si05	Si06	Si07	Si08
Nanofiber Water Retention (%)	280.0 ± 40.0	169.0 ± 23.3	196.0 ± 28.8	165.0 ± 55.0	200.0 ± 13.5
Film Water Retention (%)	2.3 ± 1.5	2.3 ± 2.6	6.6 ± 7.5	6.8 ± 3.8	1.7 ± 0.9
